# Cleveland, O., Dental Society

**Published:** 1891-10

**Authors:** 


### Cleveland, O., Dental Society.

For a purely democratic and unselfish dental society, go to Cleveland, Ohio. It was our pleasure to attend their meeting June 18th. Of course they have a president, secretary and treasurer, but these offices are not the goals of every member. Cleveland dentists are noted for their practical applications in dentistry and hence all items of interest that were introduced were practical in their nature.

The society meets at 5:30 p.m. and all business is transacted and the essayist of the evening then reads his paper and discussion follows until dinner is announced by the courteous waiter. It should have been mentioned that the meetings are held at the Hollenden Hotel in private parlors.

As the various courses are served, discussion of the paper and other topics that are selected by questions, continues till the conclusion of the dinner which lasts—well, until the subject for discussion is well ventilated and the food-stuffs become a burden to carry. The hospitality with which this society treat their guests is extravagant, but they know how to do it.

The essay of the evening was read by Dr. D. R. Jennings, and the readers of the REGISTER are to be congratulated that his paper, which is to be one of a series, has been obtained for publication. Dr. Jennings is one of those who rarely can be induced to write, and the younger men lose often the many golden grains such men possess until they do write. Dr. Jennings' paper appears in the present number of the REGISTER. The meeting usually adjourns at the close of the dinner. Would that there were many more such societies in existence. W.

